# Astaxanthin-loaded polylactic acid-glycolic acid nanoparticles ameliorate ulcerative colitis through antioxidant effects

**DOI:** 10.3389/fnut.2023.1267274

**Published:** 2023-11-09

**Authors:** Chunmei Li, Yu Zhou, Meng Yuan, Yawen Yang, Ruilong Song, Gang Xu, Gang Chen

**Affiliations:** ^1^College of Tourism and Culinary Science, Yangzhou University, Yangzhou, China; ^2^Key Laboratory of Chinese Cuisine Intangible Cultural Heritage Technology Inheritance, Ministry of Culture and Tourism, Yangzhou University, Yangzhou, China; ^3^College of Food Science and Engineering, Yangzhou University, Yangzhou, China; ^4^Institute of Comparative Medicine, College of Veterinary Medicine, Yangzhou University, Yangzhou, China; ^5^Jiangsu Co-Innovation Center for Prevention and Control of Important Animal Infectious Diseases and Zoonosis, Yangzhou University, Yangzhou, China; ^6^Department of Burn and Plastic Surgery, Northern Jiangsu People’s Hospital/Clinical Medical College, Yangzhou University, Yangzhou, China; ^7^School of Rehabilitation Science and Engineering, Qingdao Hospital (Qingdao Municipal Hospital), University of Health and Rehabilitation Sciences, Qingdao, China

**Keywords:** astaxanthin, polylactic acid-glycolic acid, antioxidation, colitis, MAPK

## Abstract

**Introduction:**

Astaxanthin (AST) is a type of carotenoid with strong antioxidant effects. However, the development and use of AST are limited by its water insolubility and low bioavailability. This study aims to investigate whether AST@PLGA can inhibit UC and reveal its possible mechanism.

**Methods:**

We tested the particle size, polydispersity index, and zeta potential of AST@PLGA. Then, the in vitro release and antioxidant capacity of AST@PLGA were tested. Finally, the mouse model of colitis was established and SOD, MDA, TNF-α, IL-1β, IL-6 and P38 as well as ERK were detected from mice.

**Results:**

Particle size, polydispersity index and zeta potential of AST @PLGA were 66.78 ± 0.64 nm, 0.247 and -9.8 ± 0.53 mV, respectively, and were stable within 14 days. Then, it was observed that the AST@PLGA nanoparticles not only maintained the effect of AST but also had a sustained release effect. Experiments in mice showed that AST@PLGA effectively reduced MDA, TNF-α, IL-1β and IL-6 levels and increased SOD levels. AST@PLGA also downregulated the protein expression of P38 and ERK. The results showed the positive protective effect of AST@PLGA in inhibiting acute colitis.

**Discussion:**

AST@PLGA nanoparticles have good stability and alleviating effect in colitis, which could be functional foods in the future.

## 1. Introduction

Inflammatory bowel disease (IBD) is a chronic inflammatory disease of the gastrointestinal tract, mainly divided into ulcerative colitis (UC) and Crohn’s disease (CD). The incidence of UC is related to environmental factors, host immune status and intestinal environment (1). Under the combined effect of genetic, environmental and psychological factors, UC always suffers from neuroendocrine dysfunction, intestinal mucosal barrier damage and immune system imbalance, resulting in local intestinal mucosal damage (2). The main clinical symptoms of UC are urgent urination, urinary incontinence, fatigue, increased stool frequency, mucus excretion, nocturnal bowel movements, abdominal discomfort, and bloody diarrhea (3). UC has become a global disease with a high mortality rate. Today, apart from colectomy, only lifelong treatment is possible. Current treatment options include aminosalicylic acid (ASA), glucocorticoids, immunosuppressants, and new biotherapies (4). Zhu et al. (5) pointed out that elevated IL-36β levels may exacerbate UC by targeting Th2 cells and limiting the development of Foxp3 Treg cells, providing mechanisms and data to support the development of IL-36β inhibitors. However, these treatments are prone to drug resistance and side effects, so UC patients urgently need new and effective drugs.

Astaxanthin (AST) is a carotenoid and the most common source of natural AST is *Haematococcus pluvialis* (6). AST has attracted great attention in scientific studies due to its strong antioxidant activity and has played a role in many fields such as cosmetics, medicine, aquaculture, etc. Compared to beta-carotene and zeaxanthin, AST is more effective in preventing excessive oxidation of unsaturated fatty acid methyl ester, and its antioxidant activity is 10 times higher than that of β-carotene and 550 times higher than that of vitamin E, respectively (7, 8). The oxygen groups in the molecular structure of astaxanthin are different from those of other carotenoids, and at both ends of the molecular structure there is a polar region that neutralizes free radicals (9). As suggested by recent studies, AST could prevent neuronal damage in Parkinson’s disease by targeting the miR-7/SNCA axis (10). Kim et al. (11) reported that AST could reduce *Helicobacter pylori*-induced superoxide dismutase 2 (SOD2) levels and SOD activity, thereby protecting gastric epithelial cells from *Helicobacter pylori* infection. Yasui et al. (12) reported that AST inhibits colitis and colitis-associated colon carcinogenesis in mice by modulating inflammatory cytokines. However, AST contains 11 conjugated double bonds, which makes it very unstable, and its high lipophilicity and heat instability limit its use in biomedicine (13). Therefore, solving this problem is the focus of current research (14).

In recent years, nanoparticles have gradually become a research focus, providing an oral delivery approach that can improve drug stability in the gastrointestinal area and improve drug-specific targeting, absorption, solubility and bioavailability (15, 16). Recent studies have confirmed that the nano-drug system is a way to solve the AST problem. Mao et al. (17) reported that encapsulation with nanostructured lipid carriers (NLC) significantly improved the physicochemical stability and potent antioxidant activity of AST. This finding showed that the properties of the nanocarrier made AST more stable and its biological activity was guaranteed. In addition, a soybean phosphatidylcholine-based liposome was used to encapsulate AST. This formulation increased the bioavailability of AST, reduced the cytotoxicity of free drugs, and protected bones from oxidative stress and inflammation through antioxidant and anti-inflammatory activities (18). Poly (lactic acid-co-glycolic acid) copolymer (PLGA) is one of the most commonly used biodegradable materials with good biocompatibility (19). It has been approved by the US Food and Drug Administration (FDA) as a safe substance for clinical use (20, 21). As a carrier, PLGA can load proteins, peptides and drugs (22). There are reports of the production of astaxanthin-containing nanoparticles for the treatment of ulcerative colitis (23). However, there are few reports of PLGA packaging of AST and is used for the treatment of colitis. Therefore, the properties and functions of AST-loaded PLGA nanoparticles (AST@PLGA) need to be further investigated.

In this study, AST@PLGA nanoparticles were prepared by the emulsion solvent evaporation method. The characterization and stability of the nanoparticles were tested for 14 days and the antioxidant activity of AST@PLGA was analyzed. Finally, the mechanism of AST@PLGA in acute colitis in mice was investigated. Our study may provide a new strategy to protect and improve the biological activity of AST.

## 2. Materials and methods

### 2.1. Materials

PLGA (lactide: glycolide 50:50; *M*_w_ 38,000–54,000) and poly vinyl alcohol (PVA, P875084) were purchased from MACKLIN (Shanghai, China). AST (SML0982) was purchased from Sigma-Aldrich (St Louis, MO, United States). Dichloromethane and ethanol were purchased from SINOPHARM (Beijing, China).

### 2.2. Preparation of AST@PLGA nanoparticles

The AST@PLGA nanoparticles were prepared by the emulsion solvent volatilization method. 10 milligrams of AST and 100 mg of PLGA were dissolved in 1 mL of dichloromethane and then entirely dissolved by ultrasound as an organic phase. The organic phase was slowly added to the 1% PVA solution drop by drop, and the organic phase of 1 mL was added to 10 mL PVA solution, and then the emulsion was obtained by ultrasound. The emulsion was stirred on a magnetic stirrer for 3–4 h in the dark at room temperature and after centrifugation at 14,000 rpm for 40 min at 4°C. The pellet was resuspended in phosphate buffer solution (PBS).

### 2.3. Transmission electron microscopy

Transmission electron microscopy (TEM) briefly, 10 μL of AST@PLGA was added to a dry copper net and stained with 2% phospho-tungstic acid. After natural drying, Tecnai T12 transmission electron microscope was used to observe and photograph the samples.

### 2.4. Particle size, zeta potential, polydispersity index, and stability

Fifty microlitre of AST@PLGA was diluted to 1 mL with PBS, and then placed in a particle size or potential sample cell. A Malvern ES90 particle analyzer was used to measure the average particle size, polydispersity index (PDI), and zeta potential of the nanoparticles using dynamic light scattering technology. The 14 days stability measurement was based on the experimental method of Elmowafy et al. (24) with some modifications. AST@PLGA nanoparticles were packaged separately and stored at 4°C in the dark for 14 days and the suspension medium used during storage was PBS. Particle size, PDI, and zeta potential of the nanoparticles were determined using dynamic light scattering.

### 2.5. Drug loading

AST was added to the dichloromethane solution. The maximum absorption wavelength of the solution was measured using an EnSight multimode plate reader (PerkinElmer, Germany). The absorbance of different contents of the AST solution was detected at the maximum absorbance wavelength, and the standard curve was constructed. The solution of AST@PLGA nanoparticles was lyophilized and dissolved in 1 mL of dichloromethane, the absorbance was measured, and the content of AST was calculated according to the standard curve. The following equation (25) was used to obtain the drug loading:


$$DL=\frac{\mathrm{Weight}\;\mathrm{of}\;\mathrm{encapsulated}\;AST}{\mathrm{Weight}\;\mathrm{of}\;\mathrm{total}\;\mathrm{nanoparticles}}$$

### 2.6. *In vitro* release study

The *in vitro* release rates of AST and AST@PLGA were determined by dialysis (26). Free AST and AST@PLGA were poured into dialysis bags (8,000–14,000 Da). The dialysis bags were then placed in a PBS buffer containing 0.1% Tween-80, kept at 37°C, and slowly stirred at a speed of 150 rpm/min for 8 h in an air bath constant temperature oscillator (Shanghai Pingxuan Scientific instrument Co., Ltd., China). Two millilitre samples in the dialysis bag were collected at predetermined intervals (0.5, 1, 2, 4, 8 h). The absorbance of each sample was measured to estimate the percentage of drug release.

### 2.7. Antioxidant activity

The 1,1-diphenyl-2-picryl-hydrazyl (DPPH) radical scavenging rate was determined according to the method of Yang and Li (27) and modified. First, The DPPH was diluted to 0.1 mM with 99.5% absolute ethanol, and different concentrations of AST and PLGA@AST were diluted by DMSO and PBS. Then the 100 μL sample solution and 50 μL of DPPH solution reacted for 30 min at room temperature in the dark, and the absorbance at 515 nm wavelength was measured as A_1_. Under the same conditions, 50 μL 99.5% absolute ethanol and 100 μL sample were measured as background control A_2_. Fifty microlitre of DPPH solution was added to 100 μL of PBS, and the absorbance was measured as blank control A_0_. The DPPH radical scavenging rate was calculated as follows: DPPH radical scavenging rate (%) = [1 − (A_1_–A_2_)/A_0_] × 100%.

The hydroxyl radical scavenging rate was determined by the method of Barreto et al. with modification (28). Fifty microlitre of 2.25 mmol/L FeSO_4_ aqueous solution, 50 μL of 9 mmol/L salicylic acid methanol solution, and 50 μL of sample solutions of different solubility were added in sequence to the 96-well plate, then 50 μL of 8.80 mmol/L H_2_O_2_ methanol solution was added to start the reaction. After 30 min of reaction at 37°C, the absorbance A_1_ was measured at 510 nm wavelength. The absorbance was measured when the sample was replaced by methanol was A_2_. The absorbance was measured when the salicylic acid-methanol solution and the H_2_O_2_ methanol were replaced by methanol was A_0_. The hydroxyl radical scavenging rate was calculated as follows: Hydroxyl radical scavenging rate (%) = [1 − (A_1_-A_2_)/A_0_] × 100%.

The determination of ferric iron-reducing capacity was based on the method of Bouabid et al. (29) with some modifications. First, the Ferric ion reducing antioxidant power (FRAP) solution was prepared by mixing 25 mL acetate buffer (300 mM), 2.5 mL 2,4,6-tripyridyl triazine (TPTZ) (2 mM), and 2.5 mL FeCl_3_·6H_2_O (20 mM). Then 5 μL sample solution, 150 μL FRAP working solution, and 15 μL distilled water were added successively to the 96-well plate, and the absorbance at a wavelength of 595 nm wavelength was determined after 10 min at 37°C.

### 2.8. Animals and treatments

Specific pathogen-free (SPF) male C57BL/6 mice were obtained from the Comparative Medicine Centre of Yangzhou University and the mice were 7 weeks old. The room was maintained under a light: dark (12 h: 12 h) cycle, and the temperature was controlled at 25°C. Mice were provided with deionized water and food *ad libitum*. The animal experiments were approved by the Jiangsu Administrative Committee for Laboratory Animals (The license number: SYXK (SU) 2022-0044).

Thirty-two mice were randomly divided into four groups, including normal group, DSS group (4% DSS), DSS + AST group (4% DSS + 25 mg/kg AST), and DSS + AST@PLGA group (4% DSS + AST@PLGA with 25 mg/kg AST content). Except for the normal group, the other groups were fed 4% dextran sodium sulfate (DSS) aqueous solution once every other day for 14 consecutive days. After that, the mice were administrated intragastric for 7 days, and then killed under anesthesia.

### 2.9. The levels of cytokines in serum

Mice blood was collected in centrifugal tubes. After standing for 1 h at 37°C, the centrifuge tubes were centrifuged at 3000 × g for 10 min to collect serum. The content of Interleukin-6 (IL-6), Interleukin-1β (IL-1β), and tumor necrosis factor α (TNF-α) was detected according to ELISA assay kits (Boster, United States). The content of superoxide dismutase (SOD) and malondialdehyde (MDA) was detected according to SOD kit and MDA kit (Beyotime, China).

### 2.10. Histopathologic analysis of colon tissue

About 2 cm of fresh intestine was flushed and fixed in 4% paraformaldehyde solution. The intestines were buried in paraffin, and then sliced for 4–5 μm sections. Finally, the sections placed on a slide were stained for hematoxylin-eosin (HE) and immunohistochemical analysis.

### 2.11. Western blot

After washing with precooled PBS, the samples were lysed with RIPA buffer (Applygen, Beijing, China), including protease and phosphatase inhibitor cocktail (New Cell Molecular Biotech, Suzhou, China). The solution was centrifuged at 12,000 × g for 10 min at 4°C. Total protein concentration in the supernatatnt was measured using bicinchoninic acid (BCA) protein assay kit.

Protein samples were added to respective channels, separated by polyacrylamide gel electrophoresis, and electro-transferred to the polyvinylidene difluoride membrane (Millipore, Germany). After sealing with 5% skim milk for 90 min, the membrane was incubated with specific primary antibodies overnight at 4°C, then the secondary antibody incubation for 1 h. ECL chemiluminescence reagent (New Cell Molecular Biotech, Suzhou, China) was added to the image by the chemiluminescence imaging analysis system (Alpha, United States). Western Blot images were analyzed by Image J software.

The primary antibodies include Phospho-P38 MAPK (Thr180/Tyr182), P38 MAPK (D13E1), Phospho-P44/42 MAPK (ERK1/2), P44/42 MAPK (ERK1/2), Anti-rabbit IgG, HRP-linked Antibody (7074S), Anti-mouse IgG, HRP-linked Antibody (7076S), β-Actin. All antibodies above were acquired from Cell Signaling Technology.

### 2.12. Statistical analysis

All results were analyzed by the software SPSS 26, and data were presented as mean ± SD. *p*-values <0.05 were regarded as statistically significant (^*^p < 0.05, ^**^p < 0.01, and ^***^p < 0.001). The other statistical dates were calculated by GraphPad Prime 9.

## 3. Results

### 3.1. Characterization of AST@PLGA nanoparticles

**Figure 1:**
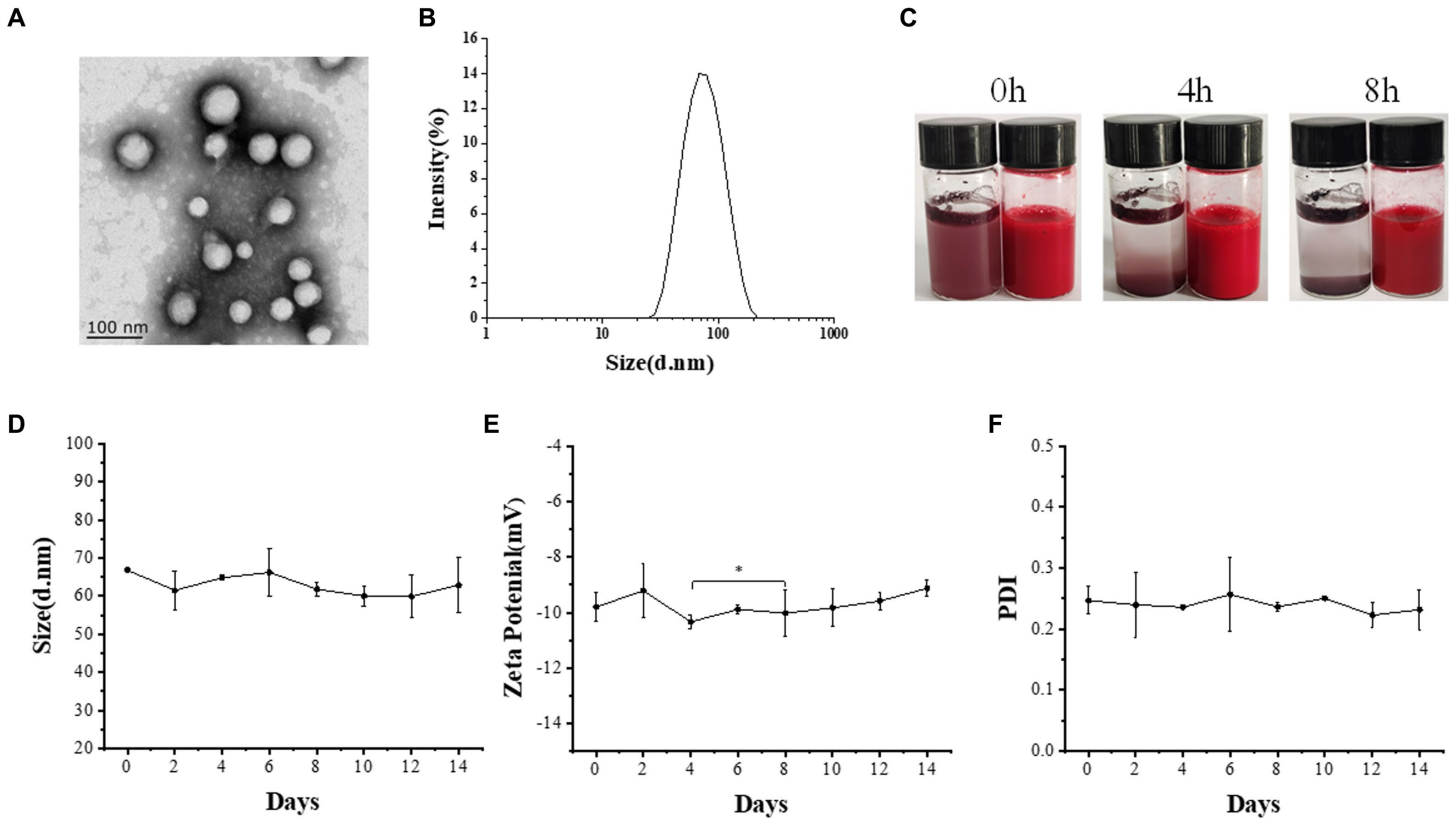
Characterization and stability of AST@PLGA nanoparticles. **(A)** TEM images of AST@PLGA nanoparticles. **(B)** The size of AST@PLGA nanoparticles. **(C)** Water solubility of AST and AST@PLGA nanoparticles. **(D–F)** Particle size, potential, and PDI of 14 days stability of AST@PLGA nanoparticles. *p*-values <0.05 were regarded as statistically significant (^*^p < 0.05, ^**^p < 0.01, and ^***^p < 0.001).

The TEM image of AST@PLGA is shown in Figure 1A. The AST@PLGA nanoparticles had a regular spherical shape. The particle size was 66.78 ± 0.64 nm and the PDI was 0.247 ± 0.02, which proved that the PDI was uniformly distributed in an aqueous solution (Figure 1B). The physical stability, dispersibility and *in vivo* properties of nanocarrier dispersions depend on the surface charge of the nanoparticles (30). The potential of AST@PLGA was −9.8 ± 0.53 mV. The AST@PLGA nanoparticles change the solubility in water. As shown in Figure 1C, AST was insoluble in water, while AST@PLGA showed water solubility after 0, 4, and 8 h. Therefore, we investigated the stability of the AST@PLGA aqueous solution in the refrigerator at 4°C for 14 days. As shown in Figures 1D–F, the particle size, potential and PDI of the AST@PLGA aqueous solution were not significantly different from those of the first day, namely the distribution and uniformity of AST@PLGA were stable in 14 days.

### 3.2. The drug loading of AST@PLGA and *in viro* release study

**Figure 2:**
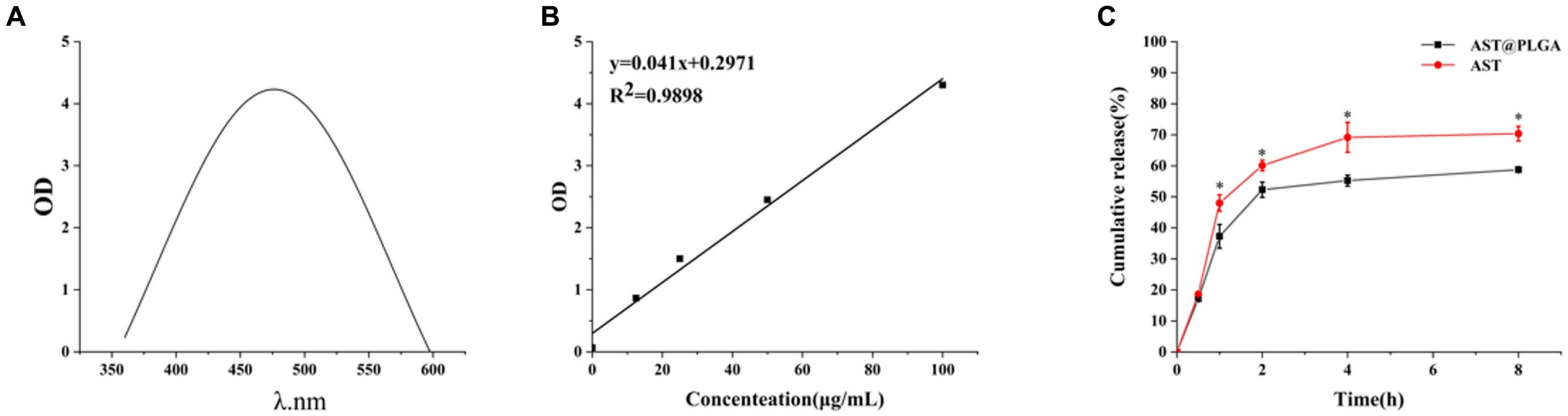
**(A)** UV–vis spectrum of AST. **(B)** Standard curve of AST. **(C)** *In vitro* release profiles of free AST and AST@PLGA. *p*-values <0.05 were regarded as statistically significant (^*^p < 0.05, ^**^p < 0.01, and ^***^p < 0.001).

Figures 2A,B showed that AST was scanned at a specific concentration using an enzyme meter. AST was found to have a maximum absorption wavelength of 480 nm. When adjusting the absorption of different AST concentrations at 480 nm, the linear regression equation for AST resulted in *y* = 0.041*x* + 0.2971. Using this equation, the drug loading rate for AST@PLGA was calculated to be 6.89 ± 0.05%. Additionally, Figure 2C showed the *in vitro* release curves for AST and AST@PLGA. The study found that AST coated with PLGA can retain the drug more efficiently. The data showed that the amount of drug released by the AST group was 60.1 ± 1.73% at 2 h and 70.3 ± 2.36% at 8 h. For AST@PLGA, the highest release rate was observed within 1 h, which accounted for 37.2 ± 3.82% of the total. This could be due to the loss of weakly adsorbed AST molecules on or near the surface of AST@PLGA (25). The release rate of AST@PLGA progressively slowed between 1 and 4 h; However, drug release persisted. After 8 h, the amount of substance released was 58.7 ± 0.81%. The total release of AST@PLGA was lower than that of AST, indicating that AST@PLGA achieved sustained release. The results showed that the PLGA vector could successfully control the release of AST, ensuring a continuous supply of the substance to the body and prolonging its effect (31).

### 3.3. Antioxidant activity *in vitro*

**Figure 3:**
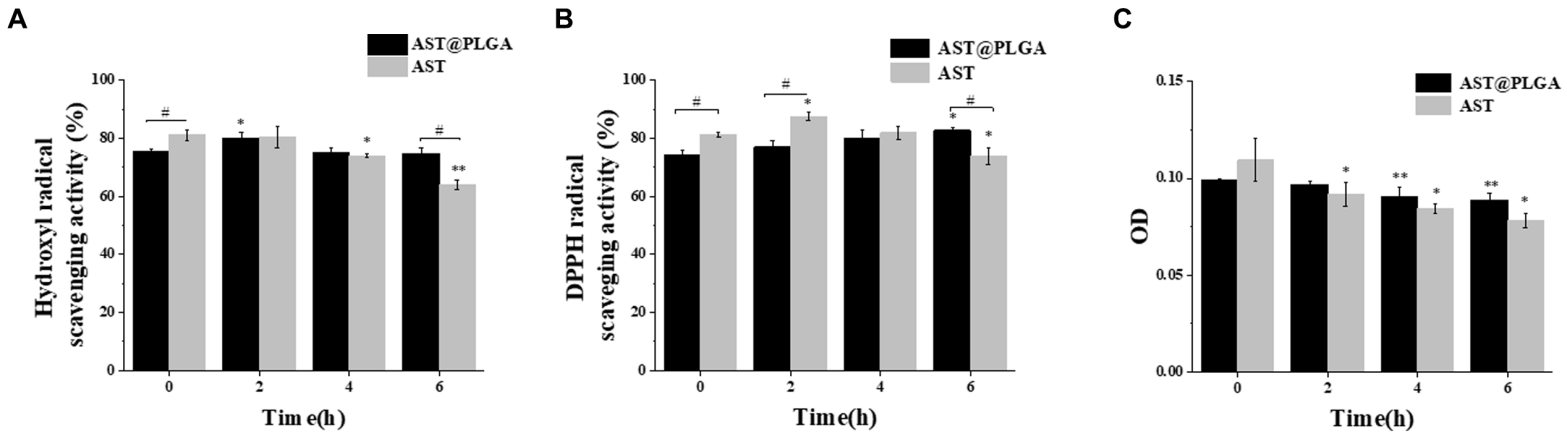
Comparison of antioxidant activity between AST@PLGA nanoparticles and AST *in vitro*. **(A–C)** Antioxidation under time gradient. (The concentration of AST and AST@PLGA is 100 μg/mL) **(A)**: hydroxyl radical scavenging rate, **(B)**: DPPH radical scavenging rate, **(C)**: reduction and antioxidation ability of trivalent iron. *p*-values <0.05 were regarded as statistically significant (^*^p < 0.05, ^**^p < 0.01, and ^***^p < 0.001), compared with the 0 h group ^#^*p* < 0.05.

AST was known for its potent antioxidant activity, so we first determined the antioxidant activity of AST@PLGA *in vitro*. Hydroxyl radicals can age the human body and cause various diseases. Therefore, scavenging hydroxyl radicals was important for human health (32). As shown in Figure 3A, the flushing rates of AST and AST@PLGA decreased with time, and the flushing rates of AST@PLGA were higher than those of AST at 4 and 6 h. DPPH was a widely accepted stable radical tool for estimating the radical scavenging activity of antioxidants (33). The DPPH radical scavenging activity of AST was higher than that of AST@PLGA in 4 h. However, after 6 h, the effect of AST@PLGA was more remarkable (Figure 3B). The reduction and antioxidation ability of trivalent iron was a typical single electron transfer method. Trivalent iron ion complex (Fe^3+^) with iron ion complex (Fe^2+^) was used to measure total antioxidant activity (34). As shown in Figure 3C, the trivalent iron reduction and antioxidant capacity of AST and AST@PLGA changed with time. The reduction of trivalent iron and antioxidant capacity of AST were weaker than that of AST@PLGA after 2 h. Of the three tests used, the FRAP, DPPH, and hydroxyl radical scavenging tests measure the reducing ability of molecules, the ability to provide hydrogen or electrons, and the ability to scavenge ROS, respectively. Although there were different mechanisms, these assays were highly correlated when used to measure antioxidant activity (35, 36). In short, AST still exhibited strong antioxidant activity after coating with PLGA. The antioxidant capacity of AST was slightly higher than that of AST@PLGA in the short term. However, the effectiveness of AST@PLGA was more sustained over time. This may be due to the encapsulation of AST in the particles by PLGA, thereby delaying its release. Compared to the unstable structure of AST, PLGA-coated AST could improve its water solubility, stability and bioavailability (37), which was consistent with the study in Figure 1C. The antioxidant experiments showed that PLGA nanoparticles maintained the antioxidant activity of AST for a long period of time.

### 3.4. The remission of clinical symptoms in colitis mice

**Figure 4:**
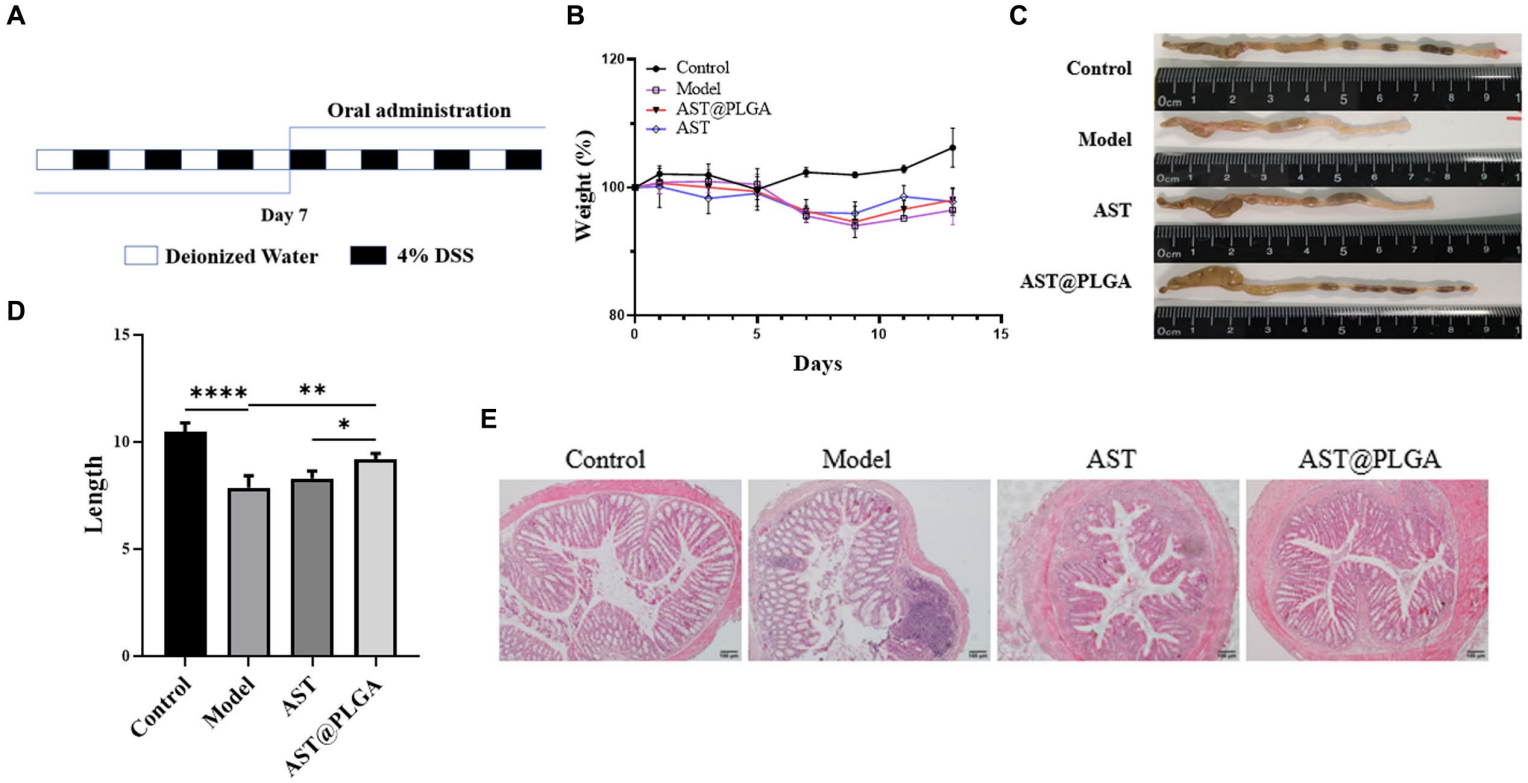
The acute colitis model building method and the effect of AST@PLGA in mice. **(A)** A schematic diagram of model process. **(B)** The changes in body weight among different groups. **(C,D)** The changes in mice colon length and shape. **(E)** The histopathological changes of mouse colon tissue. *p*-values <0.05 were regarded as statistically significant (^*^p < 0.05, ^**^p < 0.01, and ^***^p < 0.001).

AST can reduce inflammation in tissues and organs and is essential in combating intestinal inflammation and stomach ulcers caused by *Helicobacter pylori* (38). Studies have shown that AST can reduce DSS-induced weight loss, inflammatory infiltration and goblet cell depletion (14). As shown in Figures 4A,B, the body weight of mice gradually decreased after DSS water exchange, and the body weight of AST group and AST@PLGA group gradually increased after drug intervention. The length of the colon was 10.48 ± 0.42 cm in the control group, which decreased to 7.86 ± 0.56 cm in DSS-treated mice. AST@PLGA protects colon length better than AST (*p* < 0.05) (Figures 4C,D). Features of colitis include colonic mucosal injury, epithelial changes, loss of goblet cells, destruction of crypt structure, and inflammatory cell infiltration (39). In the control group, there was no obvious abnormality of the colonic mucosa and no inflammatory cell infiltration in the intestine. In the model group, while the colorectal epithelial mucosa was severely damaged, crypt branching, distortion, atrophy, goblet cell loss, inflammatory cell infiltration and other obvious phenomena occurred, there was some tissue fibrosis accompanied by free intestinal gland cells, which followed the symptoms of UC. In other groups there were varying degrees of infiltration of inflammatory cells, which were less severe than in the model group. The structure of the colon cavity in the AST@PLGA and AST groups was more expected. The results showed that DSS successfully induced UC in mice, and AST could alleviate the intestinal tissue damage of mice caused by DSS-induced UC. The ameliorative effect of AST and AST@PLGA on intestinal tissue damage was better than that of the DSS model group (Figure 4E). The results of body weight, colon length and intestinal tissue sections showed that both AST and AST@PLGA could inhibit colitis in mice, and the effects of AST@PLGA were more obvious than that of AST.

### 3.5. Effects on serum oxidative stress and tissue inflammatory cytokines secretion

**Figure 5:**
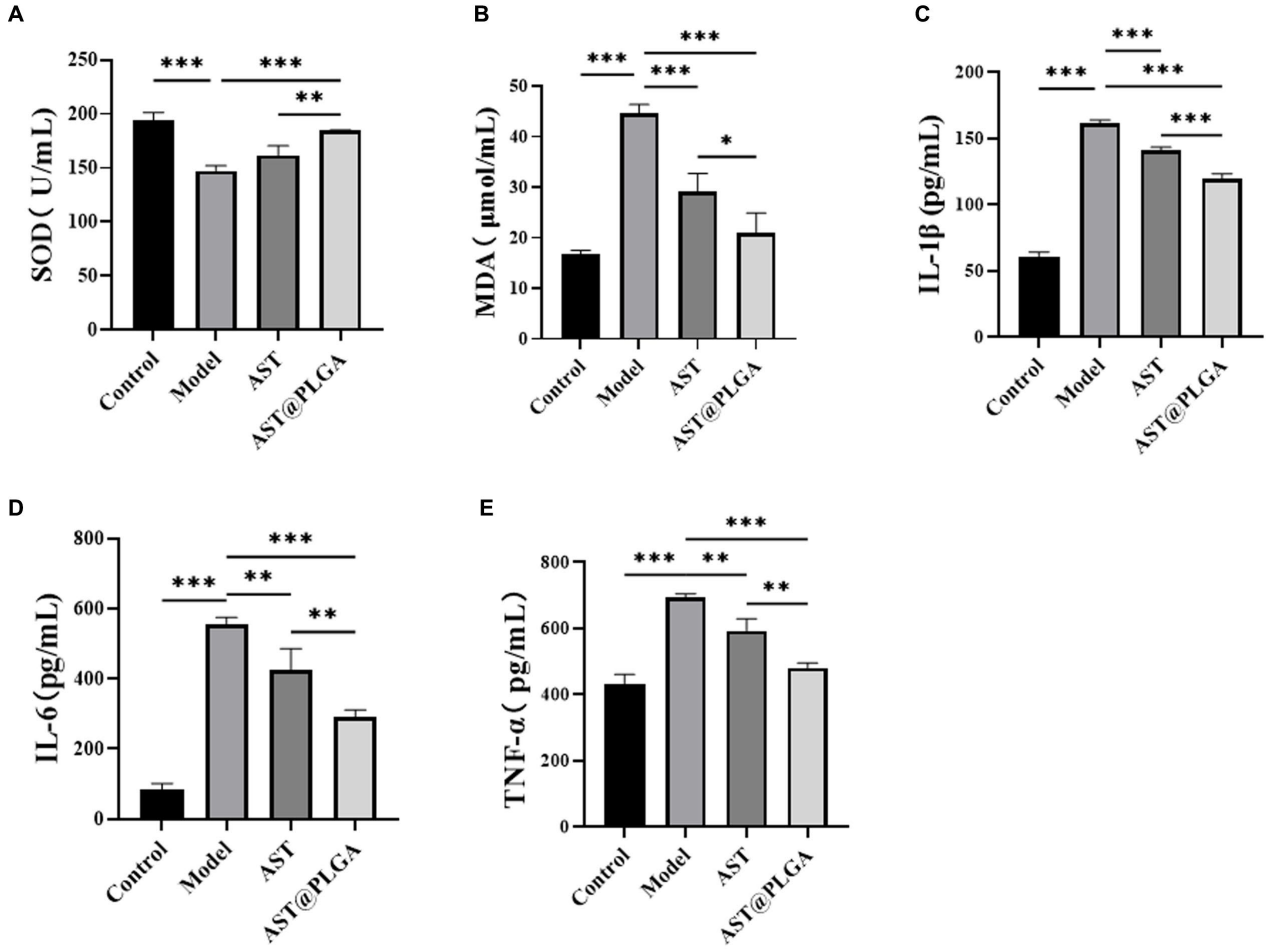
Changes of serum indexes in mice. **(A,B)** The content of SOD and MDA. **(C–E)** The content of proinflammatory cytokines IL-1β, IL-6 and TNF-α. *p*-values <0.05 were regarded as statistically significant (^*^p < 0.05, ^**^p < 0.01, and ^***^p < 0.001).

Oxidative stress is not only a risk factor for the development of chronic inflammatory diseases such as UC, but is also associated with the pathogenesis and exacerbation of UC (40). Changes in SOD and MDA levels are essential indicators of oxidative stress *in vivo*. To further elucidate the inhibitory effects of AST@PLGA and AST on UC, the levels of proinflammatory cytokines IL-6, IL-1β, TNF-α, SOD and MDA were determined in the serum of mice. As shown in Figures 5A,B, the SOD activity was significantly reduced to 147.41 ± 4.77 U/mL (*p* < 0.001) and the MDA content was significantly reduced to 44.60 ± in the model group compared to the control group 1.79 μmol/L increased (*p* < 0.001), indicating the presence of oxidative stress levels. After treatment with AST@PLGA and AST, serum SOD activity increased to 184.41 ± 0.66 and 161.83 ± 8.77 U/mL and MDA content decreased to 21.02 ± 3.93 and 29.13 ± 3.61 μmol/L. The results of MDA assay showed that AST@PLGA and AST could inhibit oxidative stress, and the efforts of AST@PLGA were higher than that of AST. These results suggest that AST@PLGA can alleviate DSS-induced oxidative stress by regulating SOD activity and MDA levels in mice. Inflammation is one of the main triggering factors in the pathogenesis of UC, which can lead to damage to intestinal tissue. TNF-α, IL-1β and IL-6 played an essential role in the inflammatory response (41). The level of pro-inflammatory cytokines in the control group was significantly lower than in the model group (*p* < 0.001) (Figures 5C–E). AST@PLGA and AST significantly reduced the production of IL-6, IL-1β, and TNF-α (*p* < 0.01). These results indicated that AST@PLGA and AST can effectively inhibit the secretion of pro-inflammatory cytokines in DSS-induced UC in mice and the efforts of AST@PLGA were higher than that of AST.

### 3.6. Effect of astaxanthin on MAPK pathway in DSS-induced mice

**Figure 6:**
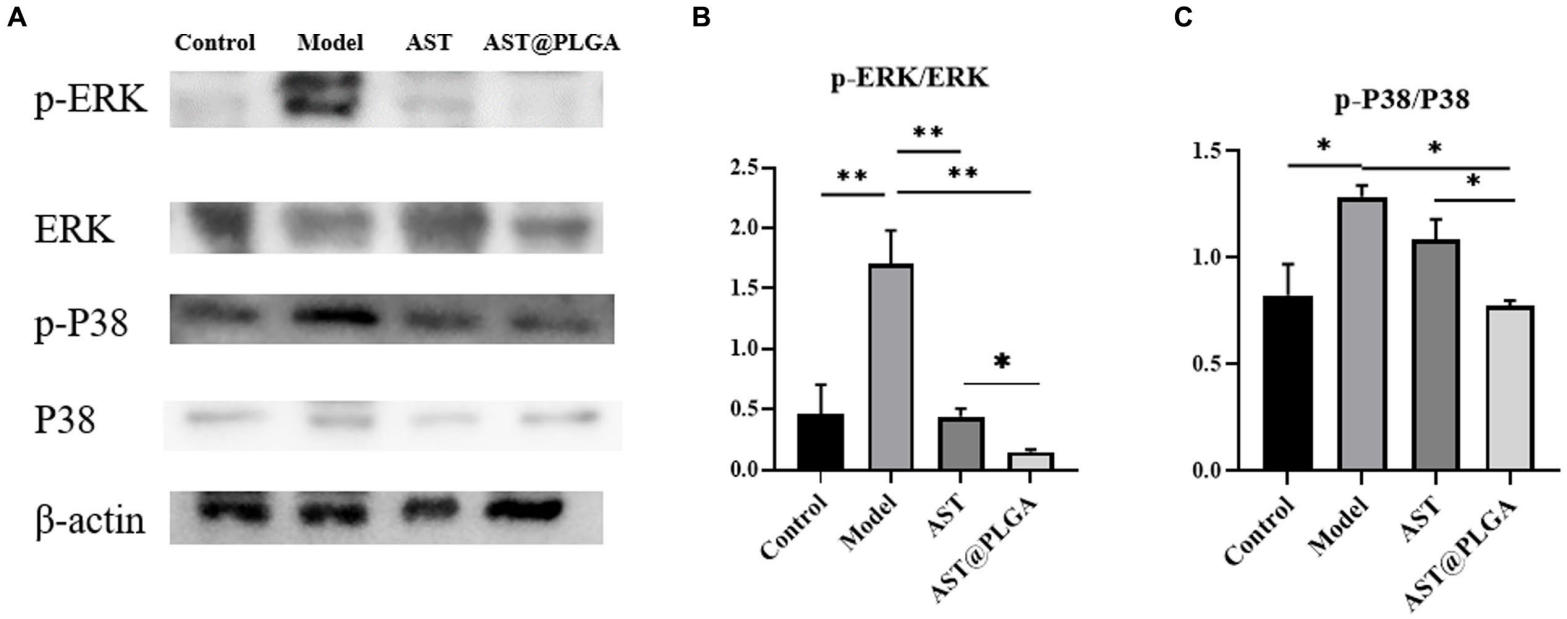
AST inhibited MAPK pathway in mice. **(A)** The representative western blotting of p-P38, P38, p-ERK, and ERK in the colon. **(B,C)** The quantification of the protein expression of p-ERK/ERK and p-P38/P38. *p*-values <0.05 were regarded as statistically significant (^*^p < 0.05, ^**^p < 0.01, and ^***^p < 0.001).

The MAPK signaling pathway (ERK, P38) is a crucial downstream signaling pathway for ROS stimulation. Several studies have shown that the MAPK pathway contributes to various biological responses in different cell types, such as Inflammation, cell growth and differentiation, and cell death and survival (42). There was increasing evidence that activation of the ERK-MAPK pathway was involved in the pathogenesis, progression and oncogenic behavior of human colorectal cancer (43). The expression of P38 and ERK-MAPK signaling pathways was detected by Western blotting, and the results are shown in Figure 6. The phosphorylation levels of P38 and ERK were significantly increased in the model group. AST@PLGA can significantly downregulate the phosphorylation levels of P38 and ERK, AST can reduce phosphorylation, and the inhibitory effect of phosphorylation of key MAPK protein is not different from AST@PLGA. In this study, AST and AST@PLGA inhibited DSS-induced MAPK phosphorylation in mouse colonic epithelial cells, and the effect of AST@PLGA is better than that of AST. An inextricable relationship between MAPK and the NF-κB signaling pathway has been reported. The MAPK pathway can activate NF-κB to regulate the expression of pro-inflammatory cytokines or mediators. As expected, AST and AST@PLGA downregulated the expression of TNF-α, IL-6, and IL-1β. Therefore, the anti-inflammatory effects of AST and AST@PLGA may be related to the activation inhibition of NF-κB and MAPK signaling pathways (44).

## 4. Conclusion

In this study, AST@PLGA was successfully prepared using the emulsifying solvent volatilization method. The nanoparticles changed their water solubility and were stable within 14 days. The AST@PLGA nanoparticles were demonstrated to exhibit sustained release through *in vitro* antioxidant and release study experiments. In a mouse model of acute colitis, AST@PLGA reduced the levels of MDA, TNF-α, IL-1β, and IL-6 and increased the levels of SOD. AST@PLGA also downregulated the protein expression of P38 and ERK. Nanomaterials had relatively small particle sizes and strong penetration, and were slowly released after astaxanthin coating, providing a new opportunity for functional food delivery.

## Data availability statement

The original contributions presented in the study are included in the article/Supplementary material, further inquiries can be directed to the corresponding authors.

## Ethics statement

The animal study was approved by Jiangsu Provincial Department of Science and Technology. The study was conducted in accordance with the local legislation and institutional requirements.

## Author contributions

CL: Funding acquisition, Writing – review & editing. YZ: Data curation, Software, Writing – original draft. MY: Writing – original draft. YY: Writing – original draft. RS: Writing – original draft. GX: Writing – original draft. GC: Funding acquisition, Writing – review & editing.
