# Supplementary material for: Astaxanthin-loaded polylactic acid-glycolic acid nanoparticles ameliorate ulcerative colitis through antioxidant effects
Source: Front Nutr. 2023 Nov 9;10:1267274. doi: 10.3389/fnut.2023.1267274 (PMC10665485; doi:10.3389/fnut.2023.1267274)
Supplement: Supplementary file 1 [file Table_1.DOCX]

https://www.jianguoyun.com/p/Dc68wK8Qm-fqCxibkZQFIAA
